# Endothelial function and germ-line *ACE I/D, eNOS* and *PAI-1* gene profiles in patients with coronary slow flow in the Canakkale population: multiple thrombophilic gene profiles in coronary slow flow

**DOI:** 10.5830/CVJA-2013-083

**Published:** 2014-02

**Authors:** Emine Gazi, Ahmet Temiz, Burak Altun, Ahmet Barutcu, Yucel Colkesen, Fatma Silan, Ozturk Ozdemir

**Affiliations:** Department of Cardiology, Faculty of Medicine, Canakkale Onsekiz mart University, Canakkale, Turkey; Department of Cardiology, Faculty of Medicine, Canakkale Onsekiz mart University, Canakkale, Turkey; Department of Cardiology, Faculty of Medicine, Canakkale Onsekiz mart University, Canakkale, Turkey; Department of Cardiology, Faculty of Medicine, Canakkale Onsekiz mart University, Canakkale, Turkey; Department of Cardiology, Faculty of Medicine, Canakkale Onsekiz mart University, Canakkale, Turkey; Department of Medical Genetics, Faculty of Medicine, Canakkale Onsekiz Mart University, Canakkale, Turkey; Department of Medical Genetics, Faculty of Medicine, Canakkale Onsekiz Mart University, Canakkale, Turkey

**Keywords:** thrombophilic genes, SNP, PAI-1 4G/5G, eNOS T-786C, ACE I/D, coronary slow flow

## Abstract

**Background:**

We examined the effects of *ACE, PAI-1* and *eNOS* gene polymorphisms on endothelial function. The genes are related to atherosclerosis and endothelial dysfunction in coronary slow flow (CSF).

**Methods:**

Thirty-three patients with angiographically proven CSF and 48 subjects with normal coronary flow were enrolled in this study. Coronary flow patterns were determined by the thrombolysis in myocardial infarction (TIMI) frame count method. Endothelial function was assessed in the brachial artery by endothelium-dependent flow-mediated dilatation (FMD). *PAI-1 4G/5G, eNOS T-786C* and *ACE I/D* polymorphisms were determined by polymerase chain reaction (PCR) amplification.

**Results:**

No difference was found between the groups regarding age, heart rate and blood pressure. Males were more prevalent among patients with CSF than control subjects (58.8 vs 29.8%, *p* = 0.009). Mean TIMI frame counts were significantly higher in CSF patients (24.2 ± 4.0 vs 13.1 ± 2.5 fpm, *p* = 0.001). FMD was significantly lower in CSF patients than in the controls (4.9 ± 6.6 vs 7.9 ± 5.6%, *p* = 0.029). TIMI frame count and FMD were found to be negatively correlated in a correlation analysis (*r* = –0.269, *p* = 0.015). *PAI-1* 4G/5G, *eNOS* T-786C and *ACE* I/D polymorphisms were similar in the two groups.

**Conclusions:**

This study showed that endothelial function was impaired in patients with CSF. PAI-1, ACE and eNOS polymorphisms were not related to CSF in our study population.

## Abstract

Coronary slow flow (CSF) was first reported in 1972 as an angiographic phenomenon, described as delayed passage of angiographic contrast agent along the coronary arteries in the absence of stenosis in the epicardial vessels.^1^ CSF is relatively rare, more frequently seen in young men and smokers with recurrent chest pain. Some cases of sudden cardiac death have been reported in patients with CSF.^2^ It was thought to be due to coronary microvascular endothelial dysfunction and diffuse atherosclerosis, although the aetiopathogenesis is unclear.^3^,^4^ Flow-mediated dilatation (FMD) is a simple, non-invasive, repetitive method for assessment of endothelial function.^5^ Impaired FMD has been reported in CSF patients.^6^

The angiotensin converting enzyme (ACE) is part of the renin–angiotensin system and plays an important role in haemostasis of the vascular wall.^7^ Regulation of the activity of ACE in both the circulation and tissues is under the control of the *ACE* gene located on chromosome 17q23. The *ACE* gene has an insertion/deletion (I/D) polymorphism in the non-coding region of the gene.^8^ Serum ACE activity is higher in subjects with deletion/deletion (D/D) alleles than in subjects with I and D alleles and is related to hypertension and cardiovascular disease.^8^,^9^ The frequency of the DD genotype and D allele was reported to be higher in SCF patients.^10^,^11^

Nitric oxide (NO) is synthesised from L-arginine by nitric oxide synthase and has an effect on endothelial relaxation.^12^ NO plays a protective role in atherogenesis, and deficiency in NO activity causes coronary spasms.^13^ A polymorphism of endothelial NO synthase (eNOS) is located on chromosome 7q35-56 and influences NO production. Nakayama *et al.* originally reported a mutation of thymidine, being replaced by cytosine at the nucleotide -786 (T-786C) gene.^14^ This polymorphism, which results in a significant reduction in eNOS gene promoter activity, is associated with hypertension, acute coronary syndrome and coronary vasospasm.^15^-^19^

Tissue plasminogen activator inhibitor 1 (PAI-1) plays an important role in endogenous fibrinolytic activity. Recent studies demonstrated that elevated PAI-1 activity was related to atherosclerosis, and was an independent predictor of coronary artery disease and myocardial infarction.^20^,^21^ The *PAI-1* gene is located on 7q21.3-22 and polymorphism of the 4G/5G gene is located in the *PAI-1* gene promoter region. The fifth guanine (G) base is inserted or deleted in the 4G sequence in the 675th base of the initial transcription point upstream. The *PAI-1* gene has three genotypes, namely, 4G/4G, 4G/5G and 5G/5G. 4G/4G allele carriers always have higher plasma PAI-1 activity than 4G/5G and 5G/5G carriers.^22^

The aim of this study was to investigate the association between ACE I/D, eNOS and PAI-1 gene polymorphisms and endothelial function, evaluated by FMD, in patients with CSF.

## Methods

A total of 33 patients with CSF (mean age 55.8 ± 10.3 years) and 48 controls (mean age 53.9 ± 11.8 years) with normal coronary arteries were enrolled in this study. Coronary angiography was performed in the cardiology clinic between January 2010 and June 2012 on patients who had an indication for elective coronary angiography due to ischaemia detected on a treadmill test and/or myocardial perfusion scintigraphy.

A complete history, findings of the physical examination, risk factors for atherosclerotic heart disease and medications were recorded. Patients who had been treated with antihypertensive drugs or those whose baseline blood pressure exceeded 140/90 mmHg were diagnosed with hypertension (HT). Diabetes mellitus (DM) was defined as fasting blood glucose levels > 126 mg/dl or the use of anti-diabetic medication. Hyperlipidaemia was defined as a total cholesterol level > 200 mg/dl and/or low-density cholesterol level > 160 mg/dl. Patients with known atherosclerotic disease, visualised coronary artery plaque in coronary angiography, peripheral artery disease, malignancy, renal and hepatic insufficiency, and chronic inflammatory disease were excluded from the study. All subjects agreed to participate in the research and the consent of the local ethics committee was obtained.

Coronary angiography was performed with a femoral approach using Judkins catheters and the contrast agent iopramide (Ultravist-370, Bayer Schering Pharma, Germany) with angiographic equipment (GE Medical Systems, Innova 2100, USA). The thrombolysis and myocardial infarction (TIMI) frame rate was 30 frames per second (fps) and angiograms were recorded on a compact disc in DICOM format. Coronary blood flow was measured quantitatively using TIMI frame count, which was determined for each major coronary artery of each subject included in the study, according to the method first described by Gibson *et al*.^23^

The left anterior descending coronary artery (LAD) is usually longer than the other major coronary arteries and for that reason the TIMI frame count of this vessel is often higher. Therefore, to obtain the corrected TIMI frame count of the LAD, the TIMI frame count was divided by 1.7.^23^

TIMI frame counts in the LAD and left circumflex (LCx) arteries were assessed in the right anterior oblique projection, and the right coronary artery (RCA) in the left anterior oblique projection. The mean TIMI frame count for each subject was calculated by adding the TIMI frame counts for the LAD/1.7, LCx and RCA and then dividing the value obtained by 3. The corrected cut-off values due to the length of normal visualisation of the coronary arteries were 36.2 ± 2.6 frames for the LAD, 22.2 ± 4.1 frames for the LCx, and 20.4 ± 3 frames for the RCA. Any values obtained above these thresholds were considered CSF.

Peripheral blood samples from CSF patients and healthy controls were used for genotyping for point mutations of *PAI-1, MTHFR* and *ACE* genes and are compared in the results. Three thrombophilic marker genes, plasminogen activator inhibitor-1 (PAI-1, rs1799889); two polymorphic regions for *MTHRF* (C677T, rs1801133 and A1298C, rs1801131), and *ACE* I/D (rs1799983) genes were analysed in the results.

## Genotyping

Peripheral blood samples containing EDTA were collected from the patients and volunteer controls after a 12-hour overnight fast. All routine biochemical tests were carried out on an autoanalyser with the Cobas 6 000 Integra (Roche Diagnostics, IN, USA) auto-analyser device using the chemiluminescence method. Venous blood was collected in 6-ml EDTA tubes for isolation of the genomic DNA and stored at –20°C. A total of 81 DNA samples from patients with CSF and controls were genotyped by real-time polymerase chain reaction (PCR) analysis.

The total genomic DNA was extracted by the MagnaPure Compact (Roche) and Invitek kit extraction techniques (Invitek®; Invisorb spin blood, Berlin, Germany). Target genes were amplified by real-time PCR, LightCycler 2.0 methods (Roche) for the CSF cohort and healthy controls. Briefly, LightCycler FastStart DNA Master HybProbes, master mix (water, PCR-grade, MgCl2, stock solution, primer mix, HtbProbe mix) and template DNA from patients and controls were used for real-time amplification for each target gene.

The amplification protocol for *MTHFR* 677C>T consisted of a denaturation step of 10 minutes at 95°C. The amplification conditions for 45 cycles were: denaturation at 95°C for five seconds, annealing at 55°C for 10 seconds, extension at 72°C for 15 seconds, melting curve step with denaturation at 95°C for 20 seconds, annealing at 40°C for 20 seconds, melting at 85°C for two seconds and the cooling step at 40°C for 30 seconds. A software program (LightCycler 2.0, Roche) was used for detection of the mutated (channel 640 at 54.5°C) and wild genotype (channel 640 at 63°C) profiles for target 677 C>T SNP analysis.

The amplification protocol for *MTHFR* 1298A>C consisted of a denaturation step of 10 minutes at 95°C. The amplification conditions for 40 cycles were: denaturation at 95°C for five seconds, annealing at 62°C for 10 seconds, extension at 72°C for six seconds, melting curve step with denaturation at 72°C for 30 seconds, annealing at 95°C for 20 seconds, melting at 40°C for one second and the cooling step at 40°C for 30 seconds. A software program (LightCycler 2.0, Roche) was used for detection of the mutated (channel 640 at 59°C) and wild genotype (channel 640 at 65°C) profiles for target 1298A>C SNP analysis.

The amplification protocol for *PAI*-1 5G/4G consisted of a denaturation step of 10 minutes at 95°C. The amplification conditions for 40 cycles were: denaturation at 95°C for three seconds, annealing at 60°C for 10 seconds, extension at 72°C for 13 seconds, melting curve step with denaturation at 95°C for 30 seconds, annealing at 40°C for one minute, melting at 85°C for two seconds and the cooling step at 40°C for 30 seconds. A software program (LightCycler 2.0, Roche) was used for detection of the mutated (4G) (channel 640 at 54°C) and wild genotype (5G) (channel 640 at 61°C) profiles for target *PAI*-1 5G/4G analysis.

The amplification protocol for *ACE I/D* consisted of a denaturation step of 10 minutes at 95°C. The amplification conditions for 45 cycles were: denaturation at 95°C for three seconds, annealing at 60°C for 10 seconds, extension at 72°C for 10 seconds, melting curve step with denaturation at 95°C for 30 seconds, annealing at 40°C for one minute, melting at 85°C for 10 seconds and the cooling step at 40°C for 30 seconds. A software program (LightCycler 2.0, Roche) was used for detection of the mutated (D, del) (channel 640 at 85°C) and wild genotype (I, Ins) (channel 640 at 93°C) profiles for target *ACE I/D* analysis.

## Echocardiography

Two-dimensional, M-mode, pulsed and colour-flow Doppler echocardiographic examinations were performed on all patients by one cardiologist with a Vivid 7 Pro echocardiography system (GE, Horten, Norway, 2–4 MHz phased-array transducer). During echocardiography, a single-lead electrocardiogram was recorded simultaneously. Data were recorded from the average of three cardiac cycles. M-mode and Doppler measurements were performed, adhering to the American Society of Echocardiography guidelines.^24^ A 10-MHz linear transducer was used for the brachial artery examination.

Endothelial function of all subjects was assessed by a single ultrasonographer blinded to the coronary flow groups. Measurements were performed in a temperature-controlled room (22°C) in the morning and after eight to 12 hours of a fasting period. Ingestion of substances that might have affected measurements, such as caffeine, high-fat foods and vitamin C was not allowed for 12 hours before the study. Any vasoactive medication was discontinued at least five serum half-lives before the brachial studies.

The right brachial artery was imaged above the antecubital fossa in the longitudinal plane. Upon acquiring an appropriate image, the surface of the skin was marked. The arm and the ultrasound probe were kept at the same position by the ultrasonographer during the entire study. The diameter of the brachial artery was measured from longitudinal images in which the lumen–intima interface was visualised on the anterior and posterior walls at end-diastole (onset of the R wave on the electrocardiogram), and the mean of the three highest measurements from five consecutive cardiac cycles was taken.

After the basal lumen diameter and blood flow were noted at rest, a sphygmomanometer cuff was placed on the forearm and the cuff was inflated to 250 mmHg for arterial occlusion. After five minutes, the cuff was deflated and the lumen diameter was recorded one minute later, to assess endothelium-dependent flow-mediated dilatation (FMD) This was defined as both the maximum absolute change and maximum percentage change in vessel diameter during reactive hyperaemia:

$FMD=\;\frac{\left(diameter\;of\;reactive\;hyperaemia-diameter\;of\;baseline\right)}{diameter\;of\;baseline}\;\times 100$

## Statistical analysis

All continuous variables were expressed as mean ± standard deviation and median (interquartile range). All measurements were evaluated with the Kolmogorov–Smirnov test, and the Shapiro–Wilk test was used to determine normal distribution. Comparisons of parametric and non-parametric values between the two groups were performed by means of Mann–Whitney *U*- or student *t*-tests. Categorical variables (risk factors and polymorphisms) were analysed using the chi-square test. Spearman’s correlation test was used for correlation between TIMI frame count and endothelial function.

All statistical studies were carried out with the program SPPS (version 15.0, SPSS, Chicago, Illinois, USA); *p*-values < 0.05 were accepted as statistically significant. Risk estimations for the association of SCF with the polymorphisms were calculated using odds ratios (OR) and 95% confidence intervals (CI) by comparing the genotypic combinations.

## Results

Clinical and laboratory findings of the subjects are shown in Table 1. Mean age and systolic blood pressure were similar between the two groups and all subjects were in sinus rhythm (55.8 ± 10.3 vs 53.9 ± 11.8 years, *p* = 0.456 and 126.4 ± 127.4 vs 127.4 ± 127.4 mmHg, *p* = 0.712, respectively). The TIMI frame counts for each epicardial artery were higher in patients with CSF than control subjects. Mean TIMI frame count was also significantly higher in CSF patients (24.2 ± 4.0 vs. 13.1 ± 2.5 fpm, *p* = 0.001).

**Table 1.** Clinical characteristics and laboratory parameters of csf patients and healthy controls

*Charactheristics*	*CSF (n = 33)*	*Controls (n = 48)*	p*-value*
Age (years, mean ± SD)	55.8 ± 10.3	53.9 ± 11.8	0.456
Heart rate (bpm)	69 ± 11	69 ± 8	0.989
Fasting glucose (mg/dl)	99 (79–281)	91 (72–188)	0.048
LDL cholesterol (mg/dl)	113 ± 35	120 ± 29	0.350
HDL cholesterol (mg/dl)	44 ± 13	46 ± 10	0.447
BSA (m^2^)	1.87 (1.61–2.19)	1.79 (1.47–2.28)	0.231
Male, *n* (%)	20 (58.8)	14 (29.8)	0.009
Hypertension, *n* (%)	17 (50)	25 (53.2)	0.777
Diabetes mellitus, *n* (%)	9 (26.5)	7 (14.9)	0.197
Cigarette smoking, *n* (%)	9 (26.5)	12 (25.5)	0.924
Medications, *n* (%)			
ACE inhibitor	12 (35.3)	12 (25.5)	0.656
Beta-blocker	6 (17.6)	8 (17)	0.941
Statins	11 (32.4)	4 (8.5)	0.006
Acetyl salicylic acid	21 (61.8)	12 (25.5)	0.001
TIMI frame count			
RCA	28 (16–38)	14 (4–22)	0.001
LCx	22 (11–40)	13 (8–21)	0.001
LAD	39.5 (22-56)	18 (10-34)	0.001
Mean TIMI frame count	24.2 ± 4	13.1 ± 2.5	0.001

CSF, coronary slow flow; LDL, low-density lipoprotein; HDL, high-density lipoprotein; BSA, body surface area; TIMI, thrombolysis in myocardial infarction; RCA, right coronary artery; LCx, left circumflex artery; LAD, left anterior descending artery; bpm, beats per minute.

Echocardiographic and FMD measurements of the subjects are summarised in Table 2. Left ventricular ejection fraction (LVEF) was significantly lower in patients with CSF [59 (27–76) vs 64% (28–76), I = 0.019). FMD was significantly lower in CSF patients than controls (4.9 ± 6.6 vs 7.9 ± 5.6%, *p* = 0.029). TIMI frame count and FMD were negatively correlated in the correlation analysis (*r* = –0.269, *p* = 0.015).

**Table 2.** Echocardiographic characteristics and flow-mediated dilatation in csf patients

*Clinical parameters*	*CSF (n = 33)*	*Controls (n = 48)*	p*-value*
LVEDD (mm)	48 (32–63)	47 (39–69)	0.755
LVESD (mm)	31 (23–48)	28 (20–60)	0.019
LVEF (%)	59 (27–76)	64 (28–76)	0.003
LA (mm)	38 (27–49)	36 (26–56)	0.059
E wave (cm/s)	66.3 ± 16.5	72.3 ± 17.5	0.127
IVRT (ms)	101.7 ± 22.7	96.9 ± 16.8	0.277
E/A ratio	1.06 (0.59–2.49)	1.12 (0.61–2.86)	0.973
E/E′ ratio	7 (3.8–15.4)	8 (3.6–19.17)	0.097
Peak S (cm/s)	8 (5–11)	8 (6–13)	0.077
FMD (%)	4.9 ± 6.6	7.9 ± 5.6	0.029

CSF, coronary slow flow; LVDD, left ventricular end-diastolic diameter; LVESD, left ventricular end-systolic diameter; LVEF, left ventricular ejection fraction; LA, left atrium; IVRT, isovolumetric relaxation time, FMD, flowmediated dilatation.

Genotype properties and allele frequencies were similar in the two groups. The *PAI-1* 5G allele was found to be marginally associated with the possibility of CSF, however it was not statistically significant (*p* = 0.06, OR: 2.82, 95% CI: 0.94–8.45) (Table 3).

**Table 3.** The polymorphic snps and genotype and allele frequencies of ace i/d, enos and pai -1 genes in csf patients and control group

*Gene/genotypes*	*CSF (n = 33) n (%)*	*Controls (n = 48) n (%)*	p*-value*	*Odds ratio*	*95% CI*
ACE I/D					
Ins/Ins	7 (21.22)	8 (16.6)			
Ins/Del	15 (45.45)	23 (47.9)			
Del/Del	11 (33.33)	17 (35.5)			
Alleles					
I	0.44	0.40	–	–	–
D	0.56	0.60	0.593	0.74	0.24–2.21
eNOS					
T/T	18 (54)	25 (52)			
T/C	13 (40)	19 (39.5)			
C/C	2 (6)	4 (8.5)			
Alleles					
T	0.74	0.71	–	–	–
C	0.26	0.29	0.759	0.87	0.37–2.06
PAI-1					
5G/5G	10 (30.4)	11 (22.9)			
5G/4G	18 (54.5)	21 (43.75)			
4G/4G	5 (15.1)	16 (33.35)			
Alleles					
5G	0.58	0.45	0.06	2.82	0.94–8.45
4G	0.42	0.55			

## Discussion

This study showed that *ACE, PAI* and *eNOS* gene polymorphisms were not related to CSF in our population. Brachial artery FMD was impaired in patients with CSF, and the TIMI frame count was negatively correlated with FMD.

ACE plays an important role in vascular wall haemostasis and endothelial function. The *ACE* D/D allele genotype was associated with higher serum ACE activity. Several studies have reported a relationship between the D allele and cardiovascular disease,^25^-^28^ and atherosclerosis.^29^,^30^ Kurtoglu *et al.* reported that concentrations of plasma endothelin-1 were increased and NOS were decreased in patients with CSF, as a result of microvascular vasomotor dysfunction, which may be important in this phenomenon.^31^

**Fig. 1.**
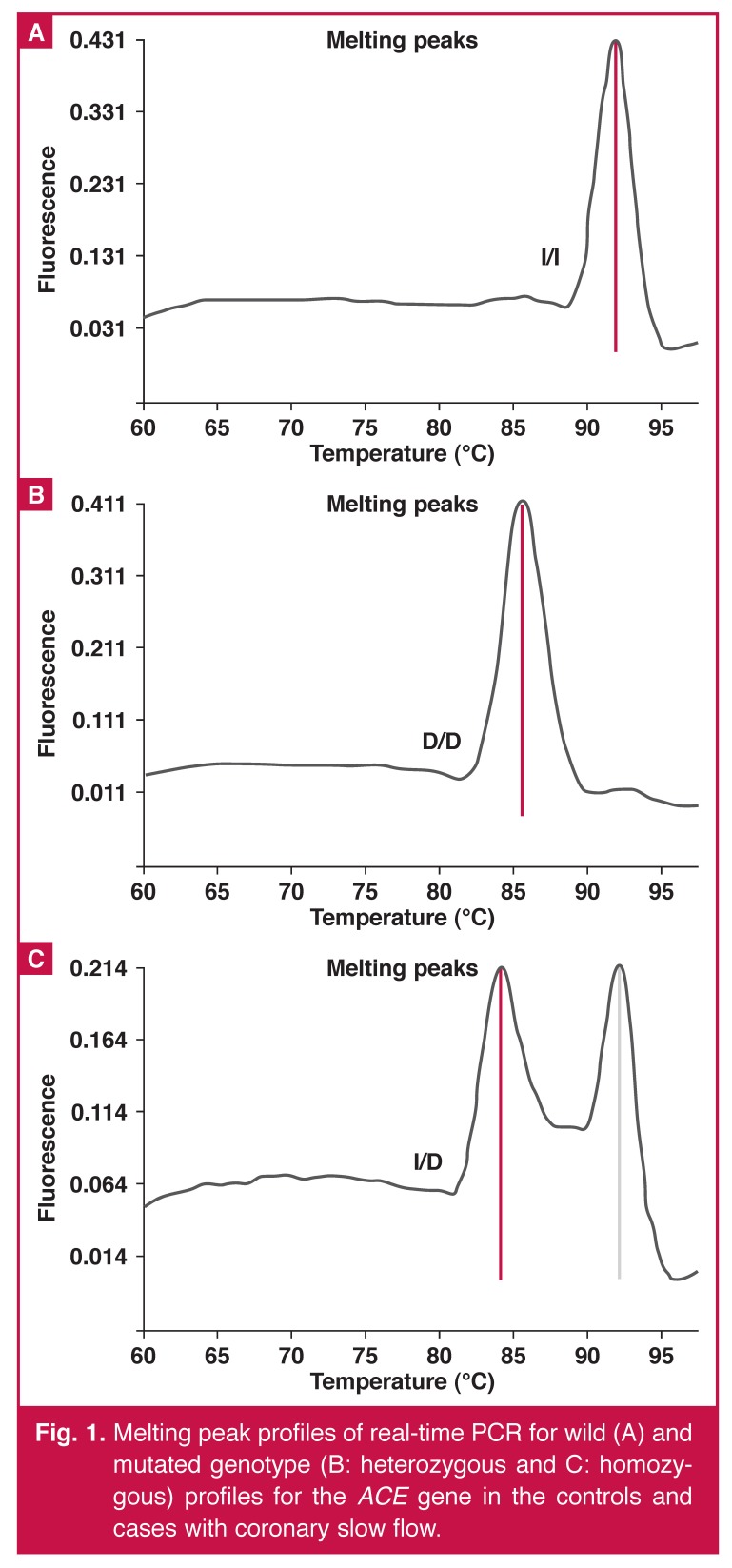
Melting peak profiles of real-time PCR for wild (A) and mutated genotype (B: heterozygous and C: homozygous) profiles for the *ACE* gene in the controls and cases with coronary slow flow.

Pekdemir *et al.* demonstrated, with intravascular ultrasonography, decreasing fractional flow reserve in the coronary arteries in patients with CSF due to diffuse atherosclerosis.^4^ Tanriverdi *et al.* reported that the *ACE I/D* polymorphism correlated with carotid intima–media thickness, which is a sign of subclinical atherosclerosis.^11^ These findings suggest that endothelial dysfunction and diffuse atherosclerosis may play a role in the pathogenesis of CSF. Yalcin *et al.* reported that the frequency of the DD genotype and D allele were higher in patients with CSF, and DD genotypes were related to a possibility of CSF.^32^ In our study we found that the D allele was not related to the presence of CSF (Fig. 1).

PAI-1 is a key regulator of the fibrinolytic process and is related to the PAI-1 promoter 4G/5G polymorphism, although it is regulated by several factors, including cytokines, growth factor and insulin.^33^-^35^ PAI-1 activity is to reduce plasma fibrinolytic activity, and poor fibrinolytic activity is related to cardiovascular events.^36^ The 4G/5G polymorphism in the promoter region of the PAI-1 gene is associated and correlated with plasma levels of PAI-1 or the response of PAI-1 to a regulator.^37^

There are few and conflicting results regarding the association of 4G allele carriers and coronary events. Some studies suggested that PAI-1 may play a role in atherogenesis due to increased PAI-1 expression, which has been demonstrated in atherosclerotic plaques.^38^ Lima *et al.* reported that plasma PAI-1 activity was higher in carriers of the 4G/4G genotype and this was correlated with atherosclerotic heart disease, as determined by coronary angiography.^39^

By contrast, Onalan *et al.* reported that the *PAI-1* 4G/4G genotype was related to a lower risk of the development of stable coronary artery disease because of the inhibitory effects of PAI on cellular migration.^40^ Likewise, some studies suggested that higher plasma levels of PAI were associated with the 4G/4G genotype, which could have been the cause of reduced plaque growth.^41^,^42^ There is no study investigating the relationship between CSF and *PAI-1* polymorphism in the literature. Our study, surprisingly, showed that *PAI-1* 4G allele carriers had a protective effect on CSF and the 5G allele was related to increasing risk for CSF.

eNOS is a regulator enzyme in the cardiovascular system for functions such as vasodilatation, inhibition of leucocyte adhesion to the endothelium, vascular small muscle cell migration and proliferation, and platelet aggregation. Reduced endothelial NO concentration is an important cause of endothelial dysfunction.^12^,^13^,^43^,^44^ The T-786C variation of the *eNOS* gene is associated with reduction in gene promoter activity and the resulting reduction in NO levels, increasing the risk for coronary spasm.^14^ Some studies have shown reduced plasma NO levels in patients with CSF.^3^,^45^

Sezgin *et al.* reported that FMD of the brachial artery was impaired and decreased plasma NO levels in patients with CSF. They concluded that endothelial dysfunction might be a cause of CSF.^46^ Nurkalem *et al.* reported an association between CSF and T-786C polymorphism of the *eNOS gene*, and a positive correlation between TIMI frame count and the C allele.^47^ In our study, T-786C genotypes were not different between CSF patients and control subjects. Our study population included only a small number of patients, which could have been the cause of the different results.

Several mechanisms, including endothelial dysfunction, diffuse atherosclerosis and small-vessel disease have been proposed as a cause of CSF.^3^,^4^ The relationship between endothelial dysfunction and atherosclerosis have been reported in previous studies.^48^,^49^ FMD is a factor in endothelial function and is correlated with carotid intima–media thickness and coronary flow reserve.^5^ Ari *et al.* reported impaired FMD of the brachial artery in patients with CSF and a negative correlation between TIMI frame count and FMD.6 In our study, FMD was impaired in patients with CSF, and negatively correlated with TIMI frame count. We found no correlation between genotyping and FMD in this study. These results suggest that endothelial dysfunction is an important process in CSF.

The most significant limitations of the present study include the small sample size; the control group was not a normal population of subjects, for ethical reasons; we could not measure inflammatory markers such as C-reactive protein, interleukins, NO and PAI-1 levels; and we could not perform intravascular ultrasonography on the patients for the determination of intimal thickening and calcification.

## Conclusion

This study shows that brachial artery FMD was impaired and the 4G allele of the *PAI-1* 4G/5G polymorphism was less prevalent among CSF patients. However, large-scale genetic studies need to be undertaken in CSF populations in order to understand the underlying mechanisms of aetiopathogenesis.
